# Les suppurations pariétales post-cesariennes au Centre Hospitalier Universitaire Yalgado Ouedraogo, Burkina-Faso: aspects epidemiologiques, cliniques, thérapeutiques et pronostiques

**DOI:** 10.11604/pamj.2019.32.35.17167

**Published:** 2019-01-18

**Authors:** Yobi Alexis Sawadogo, Evelyne Komboigo, Sibraogo Kiemtore, Hyacinthe Zamane, Issa Ouedraogo, Dantola Paul Kain, Boubakar Toure, Charlemagne Ouedraogo, Ali Ouedraogo, Blandine Thieba

**Affiliations:** 1 Unité de Formation et de Recherche en Science de la Santé Université Ouaga I Pr Joseph KI-ZERBO, Gynécologue Obstétricien, Burkina Faso; 2 Département de Gynécologie Obstétrique, CHU Yagaldo Ouédraogo, Ouagadougou, Burkina-Faso; 3 Departement de Gynécologie Obstétrique de l’Unité de Formation et de Recherche en Science de la Santé, Université Ouaga I Pr Joseph Ki-Zerbo, Burkina Faso

**Keywords:** Suppurations, paroi, césarienne, CHU/YO, Suppuration, wall, cesarean section, Yalgado Ouédraogo University Hospital

## Abstract

Les infections des sites opératoires sont fréquemment rencontrées dans les pays en développement. La césarienne étant l'une des interventions chirurgicales la plus pratiquée chez les femmes dans le monde, nous avons initié cette étude sur les aspects épidémiologiques, cliniques, thérapeutiques et pronostiques des suppurations pariétales post-césariennes dans le département de gynécologie-obstétrique du CHU Yalgado Ouédraogo en vue de réduire leur survenue. Il s'est agi d'une étude transversale à visée descriptive menée du 1^er^ avril 2015 au 30 septembre 2015 soit une période de 6 mois. Soixante-dix cas de suppurations pariétales ont été notés sur 1998 cas de césariennes soit une incidence de 3,5%. L'âge moyen des patientes était de 26,2 ans ± 6,1. Les patientes étaient majoritairement des femmes au foyer (77%). La césarienne a été réalisée en urgence chez toutes les patientes. La suppuration a été diagnostiquée surtout à la 1^ère^semaine (60%). Le germe identifié était le staphylocoque aureus dans 37,8% des cas. Une reprise chirurgicale de la paroi abdominale a été nécessaire dans 34,3% des cas. L'évolution a été favorable chez toutes les patientes. La suppuration pariétale post césarienne reste fréquente. La prise en charge nécessite parfois une reprise chirurgicale. Une meilleure identification des facteurs favorisant cette affection par d'étude plus poussée pourrait permettre de réduire de façon significative leur incidence et par conséquent améliorer le pronostic maternel.

## Abstract (en)

Surgical site infections are frequent in developing countries. Cesarean section is one of the most common surgery among women in the world. This study aims to analyse the epidemiological, clinical, therapeutic and prognostic aspects of parietal suppurations after cesarean section in the Department of Gynecology and Obstetrics at the Yalgado Ouédraogo University Hospital in order to reduce their occurrence. We conducted a descriptive cross-sectional study over a period of 6 months, from 1^st^ April 2015 to 30^th^ September 2015. Out of 1998 cases of cesarean section, seventy patients had parietal suppurations, corresponding to an incidence of 3.5%. The average age of patients was 26.2 ± 6.1 years; the patients were predominantly housewives (77%). Emergency cesarean section was performed in all patients. Suppuration was mainly diagnosed in the 1st week (60%). Staphylococcus aureus was detected in 37.8% of cases. Second abdominal wall surgery was necessary in 34.3% of cases. Outcome was favorable in all patients. Parietal suppuration after cesarean is common. Second surgery is sometimes necessary. Further studies should be conducted to better identify factors favoring this disease in order to significantly reduce their incidence and therefore improve maternal prognosis.

## Introduction

Les infections des sites opératoires sont fréquemment rencontrées dans les pays en développement [1]. La césarienne est l'une des interventions chirurgicales la plus ancienne et la plus pratiquée chez les femmes dans le monde [2, 3]. Cependant, elle présente des risques d'infection de 5 à 20 fois par rapport à l'accouchement par voie basse [4-6]. Malgré les progrès scientifiques dans tous les domaines, la césarienne n'est toujours pas une intervention anodine. En effet, elle peut être émaillée de complications qui sont entre autres, l'endométrite, les thrombophlébites, la pelvipéritonite, les infections du site opératoire avec une augmentation de la durée moyenne d'hospitalisation de 2 à 7 jours [7]. L'incidence globale des infections nosocomiales chez les femmes césarisées est estimée à 19% [7]. Si l'avènement de l'antibiothérapie, l'amélioration des techniques chirurgicales et la mise en place des systèmes de surveillance active des infections nosocomiales [7] ont permis de réduire les suppurations pariétales post-césariennes dans les pays développés, celles-ci sont encore fréquentes dans les pays en développement. Au Burkina Faso, des études spécifiques à la suppuration pariétale sont peu connues. Il nous a paru donc nécessaire de mener cette étude dans le but d'identifier les déterminants en vue d'améliorer la santé des femmes bénéficiaires de la césarienne dans le département de Gynécologie Obstétricale du CHU Yalgado Ouédraogo.

## Méthodes

L'étude s'est déroulée dans le département de gynécologie obstétrique du Centre Hospitalier Universitaire Yalgado Ouédraogo. Il s'est agi d'une étude transversale à visée descriptive. L'étude s'est étalée sur une période de 6 mois allant du 1^er^ avril 2015 au 30 septembre 2015 portant sur les parturientes admises au CHUYO et bénéficiaires d'une césarienne. Les femmes césarisées dans le département de Gynécologie Obstétrique du CHUYO et ayant présenté une suppuration post- césarienne dans les suites opératoires et qui ont accepté de participer à l'étude ont été incluses. N'ont pas été incluses les patientes ayant présenté une suppuration post-césarienne mais dont la prise en charge s'est effectuée hors du CHUYO. L'interview individuelle a été la technique utilisée pour la collecte des données. Elle a été complétée par l'exploitation des documents. Les sources documentaires étaient: le registre des admissions, les dossiers cliniques des patientes, les carnets de suivi de la grossesse, les fiches de référence et d'évacuation, le registre de césariennes et le registre du protocole opératoire. Sur le plan éthique, nous avons pris en compte le consentement des femmes dans l'inclusion. L'anonymat et la confidentialité des résultats obtenus ont été également respectés.

## Résultats

**Fréquence:** pendant la période de l'étude, nous avons enregistré 5528 accouchements avec 1998 cas de césariennes soit un taux de 36,1%. Au total 70 patientes ont présenté une suppuration pariétale. La fréquence des suppurations était de 3,5%.

**Caractéristiques générales des patientes:** la répartition des patientes selon les caractéristiques socio-démographiques a été présentée dans le Tableau 1. Trois quarts des patientes (75,7%) résidaient à Ouagadougou et aux alentours et 17 dans les autres provinces.

**Tableau 1:** répartition des patientes selon les caractéristiques socio-démographiques

Caractéristiques	Effectif	Pourcentage (%)
**Age (années)**		
15-19	12	17,1
20-24	15	21,4
25-29	23	32,9
30-34	11	15,7
35 et plus	9	12,9
**Niveau d'instruction**		
Non scolarisé	43	61,4
Primaire	10	14,3
Secondaire	13	18,6
Supérieur	4	5,7
Profession		
Femme au foyer	49	70,0
Elève/Etudiante	8	11,4
Commerçante	6	8,6
Fonctionnaire	7	10,0
**Situation matrimoniale**		
Mariée	52	74,2
Célibataire	16	22,9
Divorcée	2	2,9
**Parité**		
Grande multipare	2	2,9
Multipare	8	11,3
Pauci pare	23	32,9
Primipare	37	52,9

**Aspects cliniques:** les patientes étaient référées dans 77,1% (54 patientes) et les 16 autres patientes étaient venues d'elles même (22,8%). Quatorze (14) patientes soit 20% des cas avaient un terrain pathologique (10 drépanocytaires et 4 patientes séropositives au VIH). A l'examen physique, 53 patientes avaient une fièvre à l'admission (75,7%) et 17 patientes avaient une température normale (24,3%); 51,4% des patientes étaient anémiées. Le délai de la rupture des membranes était dans la majorité des cas supérieur à 12 heures (50%). Le Tableau 2 montre les données cliniques relatives à l'accouchement. Dans l'ensemble des 70 cas de suppurations, la césarienne a été réalisée en urgence et les indications étaient dominées par la souffrance fœtale aiguë. La plupart des patientes (82,9%) avait été opérée par les médecins en spécialisation de gynécologie obstétrique et 17,1% par des gynécologues. La durée moyenne des interventions était de 46,6 minutes ±18,9 minutes avec des extrêmes de 20 et 138 minutes.

**Tableau 2:** données cliniques relatives à l’accouchement

Caractéristique	Effectif	Pourcentage (%)
**Durée de la rupture des membranes à l’admission**		
0 ≤ 6h	27	38,6
6-12 h	8	11,4
≥ 12 h	35	50
Total	70	100
**Durée du travail en heures**		
0 ≤ 12	25	35,7
13-24	36	51,4
>24	9	12 ,9
Total	70	100
**Indication de la césarienne**		
Souffrance Fœtale Aiguë	24	34,3
Syndrome de pré-rupture	10	14,3
Utérus cicatriciel	9	12,9
Disproportion fœto- pelvienne	8	11,4
Pré éclampsie	6	8,6
Epaule négligée	5	7,1
Eclampsie	4	5,7
Présentation de siège	4	5,7
**Duré de la césarienne en minute**		
20 à 30	12	17,1
30 à59	51	72,9
60 à 138	7	10
Total	70	100

**Le suivi post opératoire:** les soins post opératoires étaient faits principalement par les antibiotiques, les solutés et les antalgiques. L'antibiotique sous forme générique est l'antibiotique administré en prophylaxie et en traitement curatif, relayé par voie orale avec l'association amoxicilline-acide clavulanique. Le traitement avait été bien administré chez cinquante-deux (52) patientes (74,3%) contre dix-huit (18) cas (25,7%) d'administration irrégulière.

**Diagnostic de la suppuration pariétale:** le délai moyen de diagnostic de la suppuration pariétale a été de 6,7 jours ± 2,3 jours avec des extrêmes de 4 et 13 jours. La suppuration a été diagnostiquée entre 4 et 6 jours postopératoires dans 60% des cas. La suppuration est survenue entre 7 et 9 jours dans 25,7% et après 9 jours dans 14,3% des cas. Trente-huit (38) cas de suppurations (54, 3%) sont survenus pendant l'hospitalisation et trente-deux (32) cas (45,7%) ont été découverts après la sortie. Un prélèvement du pus pour étude bactériologique et antibiogramme a été réalisé chez toutes les patientes. Le Staphylocoque aureus était le germe le plus fréquemment mis en évidence Les germes identifiés sont répertoriés dans le Tableau 3.

**Tableau 3:** répartition des patientes selon les germes rencontrés (n=45)

Germe identifié	Effectif	Pourcentage (%)
Staphylocoque epidermidis	5	11,1
Proteus mirabilis	5	11,1
Escherichia coli	7	15,6
Klebsiella Pneumoniae	3	6,7
Pseudomonas	2	4,4
Staphylocoque Aureus	17	37,8
Streptocoque	6	13, 3
**Total**	**45**	**100**

**La prise en charge de la suppuration pariétale:** en dehors des soins locaux des antibiotiques adaptés ont été prescrits. En fonction de l'antibiogramme, la triple association (amoxicilline - acide clavulanique + métronidazole) a été utilisée chez soixante-quatre (64) patientes soit 91,4% dans la prise en charge des suppurations par antibiotique ; des antibiotiques comme la ciprofloxacine et oxacilline ont été utilisés chez six (6) patientes soit un taux de 8,6%. La paroi a été reprise chez vingt-quatre (24) patientes (34,3%).

**Evolution:** la durée moyenne d'hospitalisation était de 23,8 jours ±12,4jours avec des extrêmes de 8 et 64 jours. Le séjour à l'hôpital était de moins de 20 jours dans 42,8%. Dans 57,2% la durée d'hospitalisation était de 20 jours et plus. Aucun décès maternel n'a été noté au cours de cette étude.

## Discussion

La fréquence des suppurations était de 3,5%. Malavaud S *et al.* [8] à Toulouse ont rapporté une plus faible incidence (2,9%). Par contre Lyimo FM *et al.* [9] notaient en 2013 en Tanzanie une fréquence de 4,8%. Il ressort de ce constat que l'incidence des infections post césariennes est toujours élevée dans les pays en développement. Le respect des normes de prévention des infections en milieu hospitalier contribuerait sans doute à la réduction de ces suppurations pariétales. L'âge moyen des patientes était de 26,2 ans ± 6,1 ans avec des extrêmes de 16 et 38 ans. Les patientes âgées de 25-30 ans étaient majoritairement représentées dans 32,8% des cas. Ce résultat est similaire à ceux d'autres auteurs [10, 11]. Plus de ¾ des patientes (77,1 %) étaient des femmes au foyer. Cela s'expliquerait par le faible niveau d'instruction des femmes. Par ailleurs, la suppuration aurait été favorisée par un certain nombre de facteurs qui sont connus. En effet à l'admission ¾ des femmes présentaient déjà une fièvre et près de la moitié des femmes étaient anémiées (48,6%). Les antécédents médicaux (certaines patientes étaient drépanocytaires et d'autres séropositive au VIH) ont certainement contribué. D'ailleurs le travail a été long chez 2 tiers des patientes (supérieur à 13 heures chez 64,3% des femmes). Plus la durée du travail est prolongée, plus le délai de la rupture des membranes est long; plus le nombre de toucher vaginal est élevé et plus grand est le risque d'ascension des germes du vagin vers la cavité utérine [11].

Cette infection utérine pourrait contaminer la paroi compte tenu de leur contiguïté. Toutes les césariennes ont été réalisées en situation d'urgence. La situation d'urgence est évoquée dans la littérature comme facteur favorisant les suppurations post césariennes, puisque toutes les mesures d'asepsie et d'antisepsie ne sont pas réunies [12] La durée moyenne de la césarienne était de 46,6 minutes avec des extrêmes de 20 et de 138 minutes. Plus la durée de l'intervention est longue plus le risque infectieux est élevé. Dans notre contexte, pour minimiser le risque infectieux, l'antibioprophylaxie peropératoire est systématique et ce traitement est poursuivi en post-césarienne. Toutefois cette antibiothérapie est plus ou moins standard et n'est pas adaptée à l'écologie bactérienne du milieu. Le délai moyen du diagnostic de la suppuration pariétale, était de 6,7 jours. Ce résultat est comparable à celui trouvé en Tanzanie par Nguhuni F *et al.* [13] et en Italie par Ferraro F *et al.* [14]. Les principaux germes identifiés étaient le Staphylococcus aureus (37,8%), l'Escherichia coli (15,6%) et le streptocoque (13,3%). Ces résultats concordent avec ceux du réseau de surveillance des infections du site opératoire de Paris [15] qui avait noté une prédominance de Staphylococcus aureus de l'ordre de 20% suivi de l'Escherichia coli 18%. Il est donc préférable de prescrire des antibiotiques couvrant ces germes en présence de facteurs de risque infectieux. Aucun décès maternel n'a été enregistré au cours de cette étude mais le séjour à l'hôpital a été long. Cette longue durée d'hospitalisation engendre un surcout pour les patientes et leur famille et accroit le risque d'autres types d'infection nosocomiale.

## Conclusion

Les infections du site opératoire sont fréquentes. Le terrain débile des patientes, le contexte d'urgence de la pratique de la césarienne et non-respect des normes de prévention des infections en pourraient constituer les facteurs favorisants. Une étude prospective s'avère nécessaire pour mieux préciser ces facteurs afin de contribuer efficacement à la prévention de ces infections du site opératoire.

### Etat des connaissances actuelles sur le sujet

- Les infections des sites opératoires sont fréquemment rencontrées dans les pays en développement;
- Les suppurations pariétales sont des complications graves pouvant compromettre le pronostic vital de la femme;
- La surveillance et le contrôle des infections nosocomiales a permis la réduction des infections postopératoires dans les pays développés.

### Contribution de notre étude à la connaissance

- Elle confirme la persistance des infections du site opératoire en post césarienne malgré les antibiotiques prescrits de façon systématique dans notre contexte;
- Les infections surviennent après la sortie de l'hôpital, nécessitant une bonne sensibilisation des femmes et une surveillance des pansements faits en externe;
- L'existence probable de résistance aux antibiotiques courants expliquerait ce fait, suggérant une étude.

## Conflits des intérêts

Les auteurs ne déclarent aucun conflit d'intérêts.
